# Mass Gatherings and Emerging Infectious Diseases: Monkeypox is the Newest Challenge

**DOI:** 10.1007/s44197-022-00059-z

**Published:** 2022-09-06

**Authors:** Jaffar A. Al-Tawfiq, Rana F. Kattan, Ziad A. Memish

**Affiliations:** 1 Infectious Disease Unit, Specialty Internal Medicine, Johns Hopkins Aramco Healthcare, Dhahran, Saudi Arabia; 2 Indiana University School of Medicine, Indianapolis, IN USA; 3 Johns Hopkins University, Baltimore, MD USA; 4 Division Head Inpatient General Pediatric, Pediatric Department, College of Medicine, King Saud Bin Abdulaziz University for Health Sciences, King Abdullah Specialist Children’s Hospital, Riyadh, Saudi Arabia; 5 King Saud Medical City, Ministry of Health, Riyadh, Saudi Arabia; 6 Al-Faisal University, Riyadh, Saudi Arabia; 7 Hubert Department of Global Health, Rollins School of Public Health, Emory University, Atlanta, GA USA

**Keywords:** Mass gatherings, Religious events, Hajj, Umrah, COVID-19, SARS-CoV-2, Monkeypox

Mass gathering medicine had recently emerged as a new discipline to deal with possible health consequences during mass gathering events (MGs). These events may pose a great heath impact especially with the occurrence of infectious diseases outbreaks and possible occurrence of international spread [1, 2]. With the emergence or re-emergence of new infectious diseases with a possible pandemic potential, a risk assessment needs to be conducted prior to hosting any MG event to predict, mitigate and control the possibility of any propagation of the outbreak associated with MGs.

The most feared outbreaks during MGs are those related to respiratory infections. In the past ten years, the Severe Acute Respiratory Syndrome coronavirus-1 (SARS-CoV-1) in 2002 and the Middle East Respiratory Syndrome coronavirus (MERS-CoV) in 2012 did not cause outbreaks during most studied annual MGs in the World (the Hajj) [3]. Similarly, the resent pandemic due to the SARS-CoV-2 had caused great risk of potential outbreaks at MGs leading to cancelation or delaying MGs events such as the 2020 Olympic Games and the Umrah and annual Hajj seasons 2000 [2, 4]. The SARS-CoV-2 had also resulted in the closure of the Grand Mosques in Makkah and Madinah [2]. Subsequently, a staged returning of Hajj in 2021 and 2022 with strict measures including enforced public health and social measures (PHSM) and effective vaccination expanding the numbers of attendees from 50 thousand attendees in 2021 to 1 million attendees in 2022 resulting in a successful Hajj with no reported coronavirus disease-2019 (COVID-19) cases among pilgrims, the accompanying healthcare personnel and the non-medical Hajj workers [5, 6]. The risk of occurrence of infectious disease during MGs depends on the venue (open air vs closed space), number and origin of attendees, ongoing outbreaks or epidemics in hosting country or countries of attendees and activities being conducted in these events and hence specific outbreaks might be associated with specific events. For example, excessive alcohol use and sexually transmitted infections among young attendees had been associated with musical and sporting MGs events [7, 8].

The multicounty re-emergence of the Monkeypox virus (MPXV) in early 2022 in countries outside Africa caused a global public health concern. MPXV is an enveloped double-stranded DNA virus belonging to the Poxviridae family and *Orthopoxvirus* genus which also includes the variola (Smallpox) virus. The MPXV is an emerging zoonotic disease endemic in rodent and nonhuman primates’ population in Africa with subsequent adaptation to humans with multiple cases occurring annually mainly in Africa. Early May 2022, an outbreak of MPXV had occurred in multiple countries around the globe with a total reported cases of 47,209 and 12 deaths in 92 countries and locations that have not historically reported monkeypox [9] (Fig. 1). Out of the two known virus clades present in Africa (Clade I, the central African or Congo Basin clade and Clade II, the West African clade), laboratory investigations have confirmed that this outbreak is caused by the West African clade of Monkeypox which has a case fatality ratio of 1% (while clade I has a case fatality ratio 10%). There was no established travel among those patients and had mainly occurred due to close contact, primarily sexual behavior amongst Gay, Bisexual, and Other men who have sex with men (MSM) [10]. There is increasing evidence that the current 2022 Monkeypox outbreak is being identified as a sexually transmitted infection [11, 12]. In a study from Spain, 92% of the identified patients with MPXV infections were gay men, bisexual men, and (MSM) and 8% were heterosexual men or women [12]. However, the number of MPXV infections had also increased in West and Central Africa over the last few years and a total of 443 cases had been reported in in locations that have historically reported monkeypox [9] making the risk of infections in MGS a likely event. Other possible routes of transmissions include respiratory droplets. In 2021, two cases of MPXV infection among traveler from Nigeria to the USA showed that of the 194 monitored contacts including 144 (74%) flight contacts had no secondary infection [13, 14] and this could be explained by the mandatory masking during the COVID-19 pandemic [14].

**Fig. 1:**
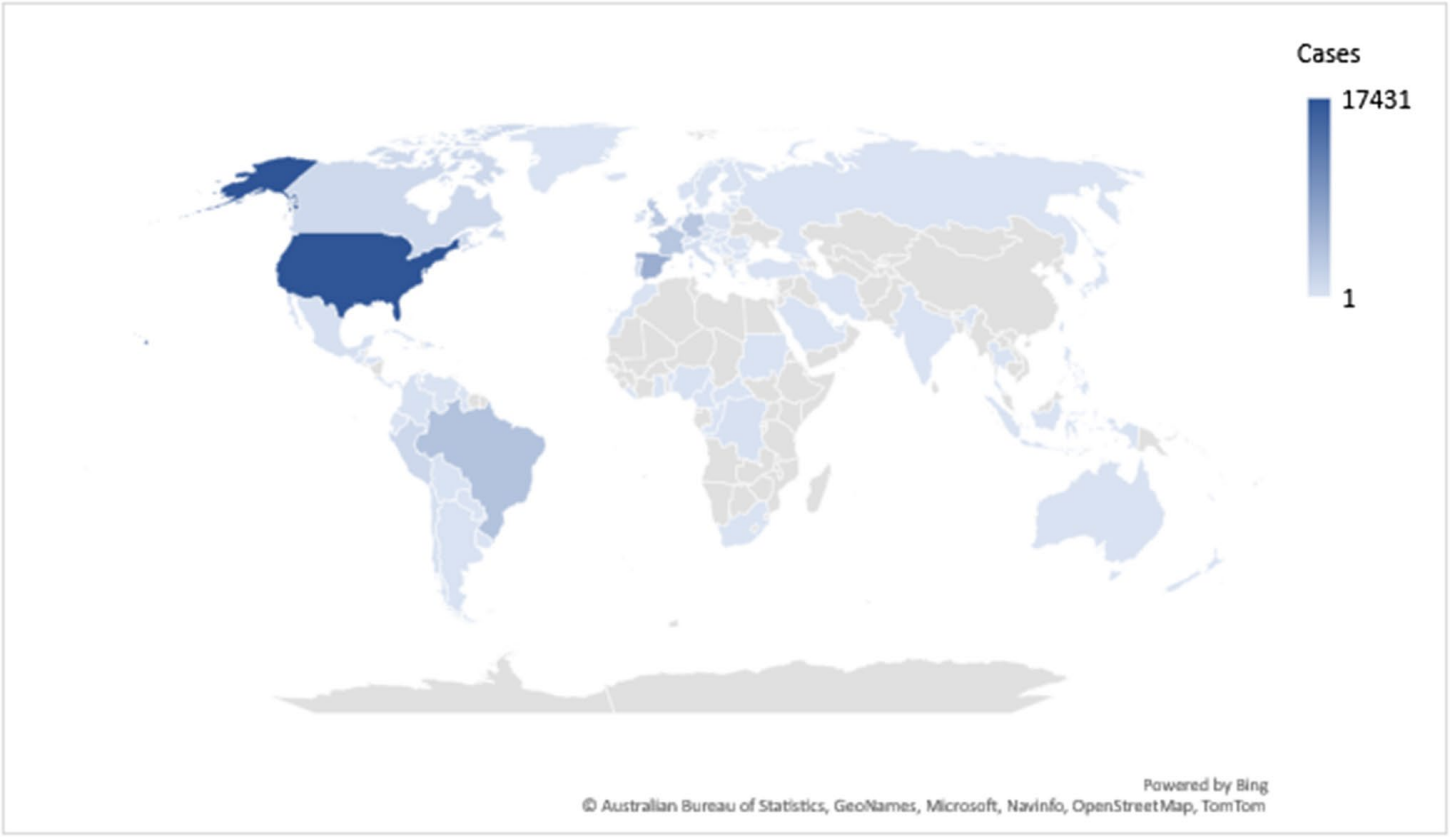
Number of Reported Monkeypox cases per Country in the 2022 Outbreak as of August 27, 2022. Data are from CDC [9]

The declaration of monkeypox as a public health emergency of international concern (PHEIC) by the world health organization (WHO) on July 23, 2022 [15] signifies the failure of public health measures taken by countries to stem the disease’s spread and stresses the urgent need to have more coordinated research and funding to expand our knowledge on best strategies for diagnosis, treatment and ramping up vaccine production for prevention of MPXV infection. This is the seventh time that WHO declares PHEIC in addition to the 2009 H1N1 influenza pandemic, poliovirus in 2014, Ebola virus in west Africa in 2014, Zika virus in 2016, Ebola virus disease in the Democratic Republic of the Congo in 2019, and COVID-19 in 2020 [16]. A recent summary of multiple documents for risk assessment, mitigation and communication during mass gatherings (Fig. 2) had been produced and summarized by the WHO Regional Office for Europe/ECDC [17]. In addition to risk assessment, host countries of MGS should include a system for thorough identification, screening, testing and contact tracing of any identified MPXV infection. A frame work had been developed by the WHO [18]. And such a system is well developed in certain MGs such as the Hajj [19], and this system could be adopted for other MGs by other organizers and countries hosting such events. There is a continued need to have strategic coordinated surveillance, appropriate testing, and epidemiological evaluation for any emerging infectious disease at all stages of MGs (before, during, and after MGS).

**Fig. 2:**
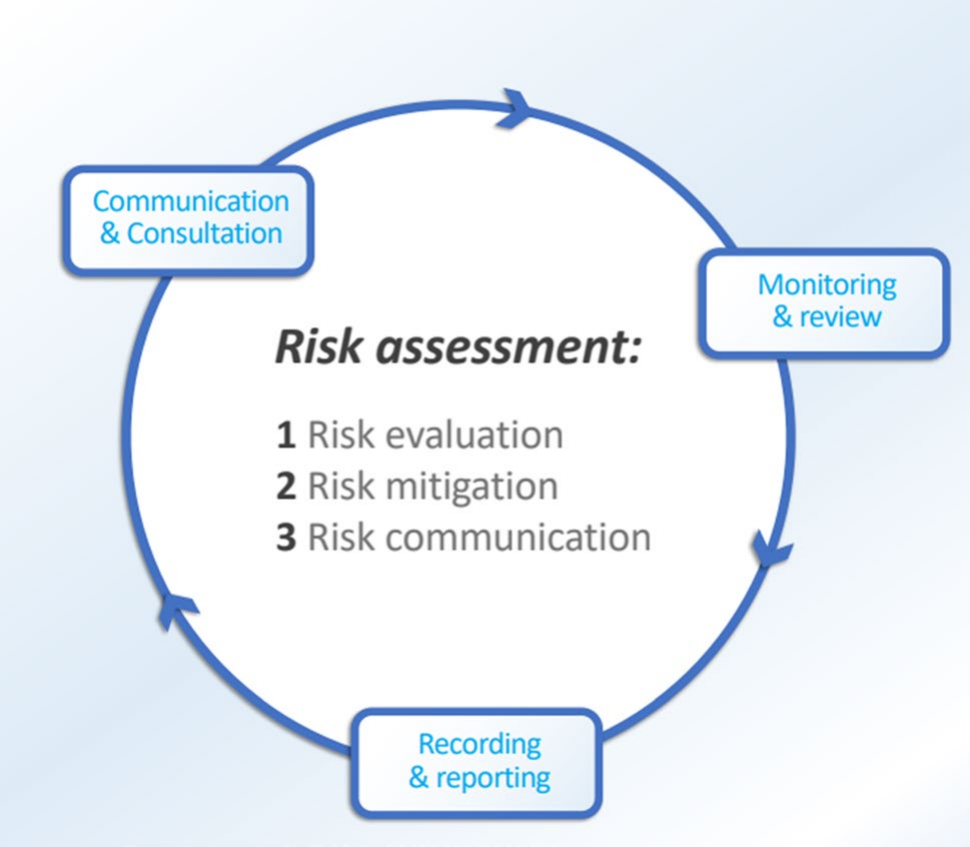
The three-step WHO Risk Assessment approach during Mass Gatherings. Adapted from the WHO [18]

The goal of the global response against MPXV is to stop the outbreak by improved surveillance and preparedness by early detection, isolation of the infected case and with case and cluster investigation and contact tracing to limit the disease transmission. These functions are much more complex to execute during MGs events, in addition to the fact that crowdedness will lead to more transmission through close contact (skin-to-skin contact) and droplet exposure via droplets. Hence the need to limit such events until this outbreak is under control and/or effective therapeutics are widely available, and vaccines can be administered to the people at highest risk of disease acquisition or complication. This is critical since more than 70% of the global population is no longer vaccinated against smallpox which provides 85% cross-protection against MPXV. Currently there are 2 smallpox vaccines available in stockpiles in the US, a replication competent vaccine (ACAM2000) and an attenuated vaccinia virus vaccine (JYNNEOS). And when it comes to therapeutic agents, two antivirals smallpox-approved drugs tecovirmat and brincidovir in addition to the non-smallpox-approved drug cidofovir. Only JYNNEOS vaccine is Food and Drug Administration (FDA) approved for monkeypox and has a much higher safety profile when compared to the ACAM2000.

Two major challenges with MPXV control during MGs event lies with the lack of ability to rapidly detect suspected cases, clusters, and sources of the infection to provide the medical care needed, isolate to prevent further spread and identify and manage all relevant contacts, especially in large uncontrolled crowds. The challenge with the suspected case identification lies in the fact that most of the newly re-merged MPXV cases present with limited anogenital rash or lesions with sparing of the face and limbs. Once the contacts of a suspected case are identified, they need to be isolated and self-monitored for symptoms and signs of MPXV for 21 days. Contacts who are pregnant, have atopic dermatitis, immunocompromised and children under 12 years of age should be offered vaccination to prevent them from getting the disease.

Although MPXV is not a new virus, causing multiple limited outbreaks in Africa and travel related clusters outside of Africa for many years, the information available about the disease are still limited, so are the availability of effective therapeutic and preventative strategies. The recent explosive re-emergence on a global level and its declaration by WHO as a PHEIC calls on all countries to take concerted collaborative efforts in collecting all relevant clinical information about the disease to better understand this infection and its rapid diagnosis and identification of the gold standard in terms of prevention, treatment, and management strategies. Until that time countries need to apply the needed risk assessments before hosting MGs to ensure the safety of the attendees and to prevent any further expansion of the pandemic.

## Data Availability

NA.

## Abbreviations

MG: Mass gathering
SARS-CoV-1: Severe acute respiratory syndrome coronavirus-1
MERS-CoV: Middle East respiratory syndrome coronavirus
PHSM: Public health and social measures
COVID-19: Coronavirus disease-2019
MPXV: Monkeypox virus
MSM: Men who have sex with men
PHEIC: Public health emergency of international concern
WHO: World health organization
FDA: Food and drug administration
